# Relationship Between Dietary Patterns and Carotid Atherosclerosis Among People Aged 50 Years or Older: A Population-Based Study in China

**DOI:** 10.3389/fnut.2021.723726

**Published:** 2021-12-01

**Authors:** Yunyun Liu, Xuena Wang, Qing Zhang, Ge Meng, Li Liu, Hongmei Wu, Yeqing Gu, Shunming Zhang, Yawen Wang, Tingjing Zhang, Magdalena J. Górska, Shaomei Sun, Xing Wang, Ming Zhou, Qiyu Jia, Kun Song, Liping Tan, Kaijun Niu

**Affiliations:** ^1^The Second Affiliated Hospital of Soochow University, Suzhou, China; ^2^Nutritional Epidemiology Institute and School of Public Health, Tianjin Medical University, Tianjin, China; ^3^Health Management Center, Tianjin Medical University General Hospital, Tianjin, China; ^4^Department of Toxicology and Sanitary Chemistry, School of Public Health, Tianjin Medical University, Tianjin, China; ^5^Nutrition and Radiation Epidemiology Research Center, Institute of Radiation Medicine, Chinese Academy of Medical Sciences & Peking Union Medical College, Tianjin, China; ^6^Center for International Collaborative Research on Environment, Nutrition and Public Health, Tianjin, China; ^7^Tianjin Key Laboratory of Environment, Nutrition and Public Health, Tianjin, China

**Keywords:** dietary patterns, carotid atherosclerosis, Chinese population, nutritional epidemiology, cross-sectional

## Abstract

**Background:** The relationship between dietary patterns and atherosclerosis is inconclusive. Usually, diets vary greatly among different regions due to cultural differences and lifestyles. Few studies to date based on a Chinese population have investigated the relationship between dietary patterns and the formation of atherosclerosis in carotid arteries. We aimed to investigate whether dietary patterns were related to carotid atherosclerosis among an adult population in Tianjin, China.

**Methods:** This cross-sectional study included a total of 2,346 participants aged 50 years or older (mean: 59.7 ± 6.29 years). Dietary intakes were assessed using a validated 81-item semiquantitative food frequency questionnaire, and factor analysis was used to identify dietary patterns. Carotid atherosclerosis was defined as a common carotid artery intima-media thickness ≥1.0 mm or plaques, or a carotid bifurcation intima-media thickness ≥1.2 mm. Multiple logistic regression models were used to explore the relationship between dietary patterns and carotid atherosclerosis.

**Results:** Three factors were determined: “health” dietary pattern (factor 1), “traditional Tianjin” dietary pattern (factor 2), and “sweets” dietary pattern (factor 3). The multivariable-adjusted odds ratios (95% CI) of carotid atherosclerosis for the increasing quartiles of the sweets dietary pattern scores in women were as follows: 1.00 (reference), 1.33 (0.91, 1.97), 1.21 (0.82, 1.79), 1.64 (1.08, 2.51) (*p* for trend <0.05). No significant difference was found between any dietary pattern and carotid atherosclerosis in men.

**Conclusion:** Greater adherence to “sweets” dietary patterns was positively related to a higher prevalence of carotid atherosclerosis in women aged 50 or older. No relationship was found between any dietary pattern and carotid atherosclerosis in men. Further prospective studies are warranted to test this finding in other populations.

## Introduction

Prospective studies have confirmed that increased carotid intima-media thickness (CIMT) and the presence of plaques are effective early markers of carotid atherosclerosis (CA), and are independent risk predictors of cardiovascular diseases (CVDs) (1–3). In addition, CA is also major comorbidity of myocardial infarction and ischemic stroke, thereby leading to premature death and disability (4). In 2020, the prevalence of increased CIMT was estimated to be 27·6% globally in people aged 30–79 years, equivalent to 1,06,670 million cases (5). The huge health burden of CA calls for efforts on effective preventative health strategies.

Modifiable lifestyle factors, such as diets and physical activity, are strongly related to chronic diseases (6). Several studies have investigated the relationship between a specific food and CA by measuring CIMT, which showed diet was one of the predictors of IMT (7–9). However, single foods or nutrients are often correlated with each other, and there are mixed interactions between foods or nutrients. Compared with the “single nutrient” approach, the analysis of the whole diet fully evaluates interactions between foods, as well as interrelationships between nutrients (10, 11). Thus, researches on dietary patterns have become increasingly popular in recent years. Studying dietary patterns is important to better understand diet and associated health outcomes and provides a practical way to translate such relationships into dietary recommendations adhered by individuals. The relationship between dietary patterns and the presence of atherosclerosis identifies strategies for preventive behavioral interventions to promote the primary prevention of cardiovascular disease.

Although several crosssectional (12, 13) and prospective cohort studies (14, 15) have investigated the relationship between different dietary patterns and CA, the results were inconclusive. Moreover, these studies included mainly Western populations, and their results may not be generalizable to Chinese adults with different lifestyles, confounding factors, and disease susceptibilities. Eating habits vary greatly between regions. A Chinese nutrition survey demonstrated that there were marked differences in the consumption of nutrients between Chinese subjects and those from Japanese, American, and Italian (16). To the best of our knowledge, only two small-scale observational studies conducted in China investigated the relationship between vegetarianism and atherosclerosis (17, 18). Little is known about the relationship between diverse dietary patterns and CA in China. Accordingly, we conducted this large-scale population study in Chinese adults aged ≥50 years with a high prevalence of CA (19).

## Methods

### Study Population

This crosssectional study was conducted on a population from Tianjin Chronic low-grade systemic inflammation and health cohort study (TCLSIH). TCLSIH is a large prospective dynamic cohort study focusing on exploring the relationship between chronic low grade systemic inflammation and the healthy condition among the population living in Tianjin, China, and is described in detail elsewhere (20, 21).

**Figure 1:**
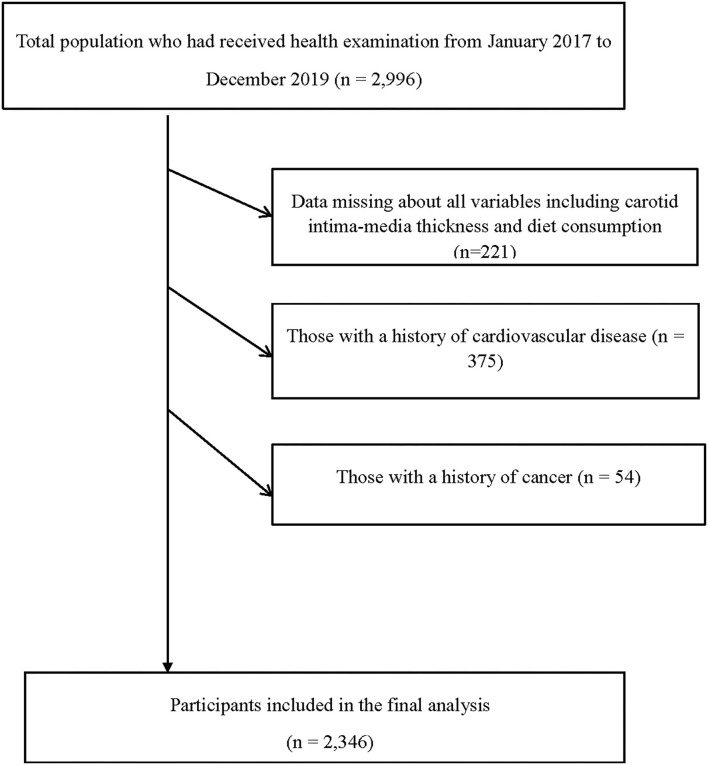
Flow diagram showing the process for the selection of eligible participants.

All participants were recruited while doing their annual health examinations at Tianjin Medical University General Hospital-Health Management. Participants completed their examinations with anthropometric parameters and biochemical blood examination and were instructed to complete the questionnaire about lifestyle, diets, current medication, history of the disease, and so on. All of them gave informed consent before enrolling. The protocol of our research was approved by the Institutional Review Board of Tianjin Medical University. In addition, the research conformed to the principle outlined in the Declaration of Helsinki. TCLSIH data from 2017 to 2019 was used to perform this crosssectional analysis. A total of 2,996 participants were enrolled in this research at first. We excluded participants who did not complete the questionnaire or who had missing variables (*n* = 221), or those with a history of CVDs (*n* = 375), or participants who had cancer (*n* = 54). Finally, there were 2,346 participants (mean ± SD age: 59.7 ± 6.29 years) remaining in the research, including 1,293 men (mean ± SD age: 59.5 ± 6.29 years) and 1,053 women (mean ± SD age: 60.1 ± 6.28 years). The process of participants selection is shown in Figure 1.

### Assessment of Dietary Patterns

Dietary consumption over the previous month was evaluated at baseline using an 81-item semiquantitative food frequency questionnaire (FFQ) with specified serving sizes that were described by natural portions or standard weight and volume measures of the servings commonly consumed in this study population. The FFQ contained seven frequencies ranging from “almost never” to “twice or more per day” for foods, and eight frequencies ranging from “almost never” to “four or more times per day” for beverages. The reproducibility and validity of the FFQ have been tested in a random sample of 150 participants living in Tianjin by comparing the data from the repeat measure ~3 months apart and 4-day weighed dietary records (WDRs) (22). Spearman's rank correlation coefficient for energy intake between two FFQs was 0.68, for food items (fruits, vegetables, meat, beverages, etc.) it ranged from 0.62 to 0.79, for energy intake by the WDRs and the FFQ it was 0.49, and for energy-adjusted nutrients (vitamin C, vitamin E, polyunsaturated fats, saturated fats, carbohydrate, etc.) by the WDRs and the FFQ, the value ranged from 0.39 to 0.72. Factor analysis was used to generate major dietary patterns and factor loadings for all 81 food items and beverages. Varimax rotation was applied for greater interpretability. With eigenvalues (>1.0) and the scree test, three factors were determined and named descriptively according to the factor loadings with absolute value ≥0.3: “health” pattern (factor 1), “traditional Tianjin” dietary pattern (factor 2), and “sweets” dietary pattern (factor 3). Dietary scores were calculated by summing the consumption of each food item weighted by its factor loading and were standardized for a mean of 0 and a SD of 1.

### Assessment of CA

Trained and certified sonographers measured CIMT of all individuals who stayed in the supine position and turned their heads 45° to the contralateral side of the artery using iU Elite (Royal Philips) with an L9-3 transducer. Sonographers scanned the far wall of the common carotid artery (CCA) and the carotid bifurcations at both the left and right carotid arteries, measuring the distance from the edge of the first echogenic line to the edge of the second echogenic line. The definition of CA is the largest CCA IMT ≥ 1.0 mm or plaques, or the largest carotid bifurcation IMT ≥ 1.2 mm (23, 24). All these measurements were conducted three times to ensure that the intrameasure and intermeasure CVs were <2.9%.

### Assessment of Other Variables

Blood samples for laboratory testing were collected in siliconized vacuum plastic tubes, and fasting blood glucose (FBG) was measured using the glucose oxidase method. Total cholesterol (TC) and triglycerides (TG) were measured using the enzymatic colorimetric methods. Lipoprotein cholesterol (LDL-C) was measured by the polyvinyl sulphuric acid precipitation method, and high density lipoprotein cholesterol (HDL-C) was measured by the chemical precipitation method. All of these were tested on the Roche Cobas 8000 modular analyzer (Roche, Mannheim, Germany). Height and weight were recorded with a standard protocol. Body mass index (BMI) was equal to weight (kg)/height (m^2^). Waist circumference (WC) was measured in standing position at the umbilical level. Systolic blood pressure (SBP) and diastolic blood pressure (DBP) were measured two times using an automatic device (TM-2655P, A&D Company, Ltd., Tokyo, Japan) in a seated position at the upper right arm. The average value was used for analysis. Socio-demographic variables like age, sex were also assessed. Information on current medication, individual history of disease, alcohol drinking status (“everyday,” “sometime,” “ex-drinker,” and “non-drinker”), and also smoking status (“smoker,” “ex-smoker,” and “non-smoker”) was obtained from questions in the questionnaire. Physical activity (PA) in the recent week was assessed by the short version of the International Physical Activity Questionnaire (25). Metabolic equivalents (METs) hours per week were calculated by the following formula: MET coefficient of activity × duration (h) × frequency (days). Corresponding MET coefficients are 3.3, 4.0, and 8.0. Total PA levels were assessed by METs hours per week (26). Depression status was measured with the Chinese version of the Zung self-rating depression scale (SDS). In this work, participants whose scores ≥ 45 were considered to have depressive symptoms (27). Hypertension was defined as having an average SBP ≥ 140 mmHg, an average DBP ≥ 90 mmHg, or as the use of antihypertension medications. Hyperlipidemia was defined as average TC ≥ 5.17 mmol/l, TG ≥ 1.7 mmol/l, LDL ≥ 3.37 mmol/l, or the use of antihyperlipidaemic medications. Diabetes was defined as having FBG levels ≥7.0 mmol/l, oral glucose tolerance test values ≥11.1 mmol/l, HbA1c ≥ 48 mmol/mol (6·5%), or a history of diabetes.

More detailed information about education levels, employment status, household income, and history of diseases was obtained from the same questionnaire. Level of education was categorized into: <college graduate, ≥college graduate. The occupation was grouped into managers, professionals, and others. For monthly household income, 10,000 ¥ was recognized as the cut-off point to divide participants.

### Statistical Analysis

We analyzed data with Statistical Analysis System 9.3 edition (SAS Institute Inc., Cary, NC, USA). Descriptive data are shown with geometric means (95% confidence interval, CI) and percentages. The different characteristics of participants according to CA status were examined using analysis of variance for continuous variables and chi-square test for variables of proportion. Dietary pattern scores were categorized into quartiles for each dietary pattern based on the distribution of the score of all participants. After testing for linearity, we examined the relationship between average dietary pattern scores' quartile and CA in multivariable logistic regression models.

The CA was a dependent variable. Model 1 included the standardized medial value of each quartile as an independent variable. In model 2, we added age, and BMI as adjusted confounding factors. In model 3, we additionally adjusted for the following potential confounders: smoking status, alcohol drinking status, educational levels, PA, employment status, incomes, total energy consumption, depression status, history of hypertension, hyperlipidemia, and diabetes. Model 4 was additionally adjusted for scores of the other two dietary patterns. Variance inflation factor (VIF) was used to assess multicollinearity among covariates. VIF exceeding 10 was a sign of multicollinearity. We calculated ORs and 95% CI. All *P*-values for linear trends were calculated using the medial value of each quartile as a continuous variable based on linear regression, and two-tailed *p*-values <0.05 were defined as statistically significant.

## Results

**Figure 2:**
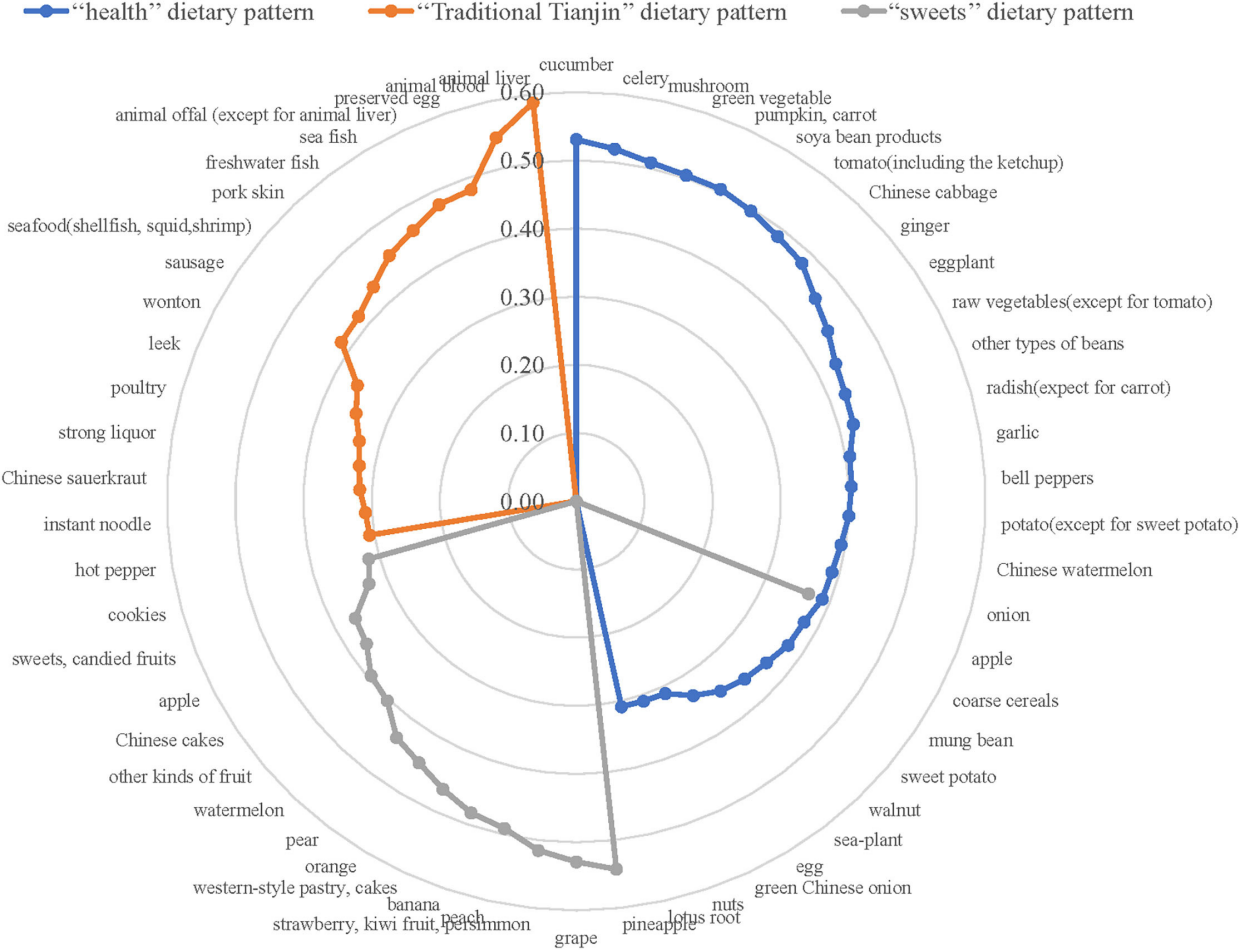
Radar graph of factor loadings characterizing dietary patterns. The blue line indicates factor loadings related to the “health” dietary pattern. The orange line indicates factor loadings related to the “Traditional Tianjin” dietary pattern. The gray line indicates factor loadings related to the “sweets” dietary pattern. Dietary patterns are described based on factor loadings with absolute value ≥0.3.

Three dietary patterns were generated by factor analysis according to factor loadings with varimax rotation. Figure 2 showed the main factors of each dietary pattern. Three factors explained 17.3% of the variance in dietary consumption (i.e., 7.31% for factor 1, 5.05% for factor 2, and 4.89% for factor 3). Factor 1 was defined as a healthy dietary pattern, which was characterized by a high intake of vegetables like cucumber, mushroom, celery, pumpkin, green vegetable, or soya bean products. Factor 2 was defined as the “Traditional Tianjin” dietary pattern, which was in a high intake of animal liver, animal blood, seafood, sea-fish, freshwater fish. Factor 3 was defined as sweets dietary pattern, which was in a high intake of fruits, cakes, western-style pastry and sweets.

A total of 2,346 adults were included in this research finally. The overall prevalence of CA in participants was 31.8%. Tables 1, 2 showed the age-adjusted difference of characteristics among men and women according to the status of CA. In men, compared with participants who did not have CA, participants with CA tended to be older and had a higher BMI, WC, TC, TG, LDL, SBP, DBP, FBG, but a lower HDL. They were also more likely to be smokers and less likely to be non-smokers. They tend to have a history of hypertension, hyperlipidemia, and diabetes (all *p* < 0.05). Otherwise, there was no significant difference for other variables. In women, compared with participants who did not have CA, participants with CA tended to be older and had a higher BMI, WC, TC, TG, LDL, SBP, DBP, FBG, but a lower HDL. Moreover, those participants are more likely to have a monthly household income >10,000 ¥ (all *p* < 0.05). Otherwise, there was no significant difference for other variables.

**Table 1:** Age-adjusted baseline characteristics of study participants by the prevalence of CA in men (*n* = 1,293)^a^.

**Characteristics**	**CA**		***P*-value^b^**
	**No**	**Yes**	
No. of subjects	548	745	-
Age (years)	57.1 (56.6, 57.6)^c^	60.7 (60.3, 61.2)	<0.0001
BMI (kg/m^2^)	25.5 (25.2, 25.8)	26.3 (26.1, 26.5)	<0.0001
WC (cm)	90.6 (89.8, 91.3)	92.5 (91.9, 93.1)	<0.001
TC (mmol/L)	4.85 (4.80, 4.90)	5.05 (5.00, 5.10)	<0.001
TG (mmol/L)	1.47 (1.40, 1.50)	1.56 (1.50, 1.60)	<0.05
LDL-C (mmol/L)	2.82 (2.70, 2.90)	3.02 (3.00, 3.10)	<0.0001
HDL-C (mmol/L)	1.23 (1.20, 1.30)	1.19 (1.18, 1.20)	<0.05
SBP (mmHg)	129.3 (127.9, 130.7)	132.2 (130.9, 133.4)	<0.01
DBP (mmHg)	82.6 (81.6, 83.5)	84.2 (83.3, 85.0)	<0.05
FBG (mmol/L)	5.42 (5.30, 5.50)	5.77 (5.70, 5.90)	<0.0001
Physical activity (MET-hour/week)	13.7 (12.2, 15.4)	13.1 (11.8, 14.4)	0.53
Total energy intake (kcal/day)	2356.2 (2288.1, 2424.4)	2441.9 (2383.8, 2499.9)	0.07
SDS score	34.4 (33.6, 35.1)	34.5 (33.9, 35.1)	0.83
Smoking status (%)			
Current smoker	34.7	39.0	<0.01
Ex-smoker	16.0	20.8	0.09
Non-smoker	49.3	40.2	<0.001
Alcohol drinking status (%)			
Everyday drinker	15.8	19.3	0.18
Sometime drinker	66.1	58.1	0.28
Ex-drinker	8.42	11.1	0.51
Non-drinker	9.62	11.5	0.59
Education level (≥College graduate, %)	44.2	40.8	0.54
Working status (%)			
Managers	45.8	41.6	0.33
Professionals	10.7	10.2	0.67
Other	38.5	46.3	0.59
Household income (>10,000 ¥ %)	40.5	30.2	0.13
Individual history of disease (%)			
Hypertension	46.9	60.7	<0.001
Hyperlipidemia	58.8	64.3	<0.01
Diabetes	7.12	16.8	<0.0001

a *CA, carotid atherosclerosis; BMI, body mass index; WC, waist circumference; TC, total cholesterol; TG, triglycerides; SBP, systolic blood pressure; DBP, diastolic blood pressure; FBG, fasting blood glucose; LDL-C, low-density lipoprotein cholesterol; HDL-C, high-density lipoprotein cholesterol; SDS, Self-Rating Depression Scale; MET, metabolic equivalent*.

b *Analysis of variance or chi-square test*.

c *Adjusted geometric mean (95% CI) (all such values)*.

**Table 2:** Age-adjusted baseline characteristics of study participants by the prevalence of CA in women (*n* = 1,053)^a^.

**Characteristics**	**CA**		***P*-value^b^**
	**No**	**Yes**	
No. of subjects	598	455	-
Age (years)	58.1 (57.7, 58.6)^c^	62.1 (61.6, 62.7)	<0.0001
BMI (kg/m^2^)	24.5 (24.2, 24.7)	25.2 (24.9, 25.5)	<0.001
WC (cm)	82.8 (82.1, 83.6)	84.2 (83.4, 85.1)	<0.05
TC (mmol/L)	5.24 (5.17, 5.32)	5.48 (5.39, 5.57)	<0.001
TG (mmol/L)	1.28 (1.23, 1.33)	1.39 (1.32, 1.45)	<0.01
LDL-C (mmol/L)	3.02 (2.95, 3.09)	3.29 (3.21, 3.38)	<0.0001
HDL-C (mmol/L)	1.49 (1.46, 1.52)	1.42 (1.39, 1.45)	<0.01
SBP (mmHg)	128.5 (127.2, 129.9)	133.7 (132.0, 135.3)	<0.0001
DBP (mmHg)	77.2 (76.4, 78.1)	80.0 (78.9, 81.0)	<0.001
FBG (mmol/L)	5.27 (5.20, 5.40)	5.47 (5.40, 5.60)	<0.01
Physical activity (MET-hour/week)	12.5 (11.1, 14.1)	13.0 (11.3, 14.8)	0.70
Total energy intake (kcal/day)	2109.4 (2046.0, 2172.8)	2148.1 (2074.9, 2221.4)	0.45
SDS score	35.6 (34.8, 36.3)	35.2 (34.4, 36.1)	0.60
Smoking status (%)			
Current smoker	2.50	5.21	0.08
Ex-smoker	0.58	0.99	0.67
Non-smoker	96.9	93.8	0.07
Alcohol drinking status (%)			
Everyday drinker	1.11	0.92	0.76
Sometime drinker	36.7	33.6	0.57
Ex-drinker	5.72	6.44	0.44
Non-drinker	56.5	59.1	0.39
Education level (≥ College graduate, %)	22.2	19.7	0.91
Working status (%)			
Managers	20.9	20.0	0.62
Professionals	10.2	6.49	0.38
Other	66.5	73.0	0.52
Household income (>10,000 ¥, %)	22.6	28.1	<0.0001
Individual history of disease (%)			
Hypertension	35.0	56.0	<0.0001
Hyperlipidemia	62.9	75.6	<0.001
Diabetes	4.35	11.0	<0.01

a *CA, carotid atherosclerosis; BMI, body mass index; WC, waist circumference; TC, total cholesterol; TG, triglycerides; SBP, systolic blood pressure; DBP, diastolic blood pressure; FBG, fasting blood glucose; LDL-C, low-density lipoprotein cholesterol; HDL-C, high-density lipoprotein cholesterol; SDS, Self-Rating Depression Scale; MET, metabolic equivalent*.

b *Analysis of variance or chi-square test*.

c *Adjusted geometric mean (95% confidence interval) (all such values)*.

Table 3 shows the main relationship between CA and dietary patterns among participants. In the male group, there was no relationship between any dietary pattern and CA. In the female group, the adjusted ORs (95% CI) of sweets dietary pattern across quartiles were 1·00 (reference), 1.33 (0.91, 1.97), 1.21 (0.82, 1.79), 1.64 (1.08, 2.51) (*p*_fortrend_ < 0.05). Female participants in the highest quartile of sweets dietary pattern scores had a 1.64-fold greater risk of developing CA than those in the lower quartiles. No relationship was found between the other two dietary patterns and CA in females.

**Table 3:** Adjusted relationships between dietary patterns to the prevalence of CA^a^.

	***Q*_1_**	***Q*_2_**	***Q*_3_**	***Q*_4_**	***P* for trend^b^**
**Males**					
Healthy dietary pattern	−0.95	−0.31	0.28	1.23	-
Total	324	323	322	324	-
No. of CA	162	186	197	200	-
Crude	1.00 (ref)	1.36 (1.00, 1.85)^c^	1.58 (1.15, 2.16)	1.61 (1.18, 2.21)	<0.01
Age- and BMI-adjusted	1.00 (ref)	1.37 (0.99, 1.90)	1.45 (1.04, 2.01)	1.35 (0.97, 1.88)	0.07
Multiple adjusted^d^	1.00 (ref)	1.30 (0.92, 1.85)	1.39 (0.96, 2.02)	1.26 (0.83, 1.91)	0.24
Multiple adjusted^e^	1.00 (ref)	1.31 (0.92, 1.85)	1.40 (0.97, 2.02)	1.24 (0.81, 1.91)	0.26
Traditional Tianjin dietary pattern	−0.61	−0.16	0.31	1.22	-
Total	324	323	322	324	-
No. of CA	189	192	173	191	-
Crude	1.00 (ref)	1.05 (0.77, 1.43)	0.83 (0.61, 1.13)	1.03 (0.75, 1.4)	0.75
Age- and BMI-adjusted	1.00 (ref)	1.23 (0.88, 1.73)	1.15 (0.82, 1.61)	1.37 (0.98, 1.93)	0.11
Multiple adjusted^d^	1.00 (ref)	1.19 (0.84, 1.69)	0.99 (0.69, 1.42)	1.07 (0.73, 1.56)	0.98
Multiple adjusted^e^	1.00 (ref)	1.20 (0.84, 1.70)	1.00 (0.70, 1.43)	1.11 (0.75, 1.62)	0.94
Sweets dietary pattern	−1.13	−0.51	0.00	0.98	-
Total	324	323	322	324	-
No. of CA	185	191	192	177	-
Crude	1.00 (ref)	1.09 (0.80, 1.49)	1.11 (0.81, 1.52)	0.91 (0.66, 1.23)	0.57
Age- and BMI-adjusted	1.00 (ref)	1.06 (0.76, 1.48)	1.10 (0.79, 1.53)	0.86 (0.62, 1.19)	0.42
Multiple adjusted^d^	1.00 (ref)	1.18 (0.83, 1.66)	1.22 (0.86, 1.73)	0.91 (0.63, 1.32)	0.73
Multiple adjusted^e^	1.00 (ref)	1.20 (0.85, 1.71)	1.24 (0.87, 1.77)	0.95 (0.65, 1.39)	0.90
**Females**					
Healthy dietary pattern	−1.10	−0.48	0.06	0.92	-
Total	264	263	262	264	-
No. of CA	103	115	111	126	-
Crude	1.00 (ref)	1.22 (0.86, 1.72)	1.15 (0.81, 1.63)	1.43 (1.01, 2.02)	0.07
Age- and BMI-adjusted	1.00 (ref)	1.06 (0.73, 1.54)	0.92 (0.63, 1.34)	1.16 (0.80, 1.67)	0.61
Multiple adjusted^d^	1.00 (ref)	1.00 (0.67, 1.48)	0.86 (0.57, 1.31)	0.95 (0.60, 1.51)	0.69
Multiple adjusted^e^	1.00 (ref)	1.04 (0.70, 1.55)	0.92 (0.60, 1.40)	1.03 (0.64, 1.66)	0.88
Traditional Tianjin dietary pattern	−0.97	−0.63	−0.30	0.27	-
Total	264	263	262	264	-
No. of CA	119	117	106	113	-
Crude	1.00 (ref)	0.98 (0.69, 1.38)	0.83 (0.59, 1.17)	0.91 (0.65, 1.29)	0.43
Age- and BMI-adjusted	1.00 (ref)	1.06 (0.73, 1.53)	0.89 (0.61, 1.29)	1.01 (0.70, 1.47)	0.83
Multiple adjusted^d^	1.00 (ref)	0.99 (0.67, 1.46)	0.84 (0.57, 1.24)	0.90 (0.61, 1.33)	0.45
Multiple adjusted^e^	1.00 (ref)	1.04 (0.70, 1.54)	0.88 (0.59, 1.29)	0.91 (0.61, 1.36)	0.45
Sweets dietary pattern	−0.74	−0.25	0.23	1.12	-
Total	264	263	262	264	-
No. of CA	105	120	109	121	-
Crude	1.00 (ref)	1.27 (0.90, 1.8)	1.08 (0.76, 1.53)	1.28 (0.91, 1.81)	0.30
Age- and BMI-adjusted	1.00 (ref)	1.40 (0.97, 2.03)	1.21 (0.83, 1.75)	1.55 (1.07, 2.25)	0.05
Multiple adjusted^d^	1.00 (ref)	1.34 (0.91, 1.98)	1.21 (0.82, 1.79)	1.65 (1.09, 2.49)	<0.05
Multiple adjusted^e^	1.00 (ref)	1.33 (0.91, 1.97)	1.21 (0.82, 1.79)	1.64 (1.08, 2.51)	<0.05

a *Multiple logistic regression analysis. BMI, body mass index; CA, carotid atherosclerosis; Q, quartile*.

b *P for the trend is calculated across quartiles*.

c *Odds ratio (95% confidence interval) (all such values)*.

d *Additionally adjusted for smoking status (categorical; current smoker, ex-smoker, or non-smoker), alcohol drinking status (categorical; everyday drinker, sometimes drinker, ex-drinker, or non-drinker), educational level (categorical: < or ≥ college graduate), working status (categorical; managers, professionals, and other), household income (categorical: ≤ or >10,000 Yuan), physical activity (continuous; MET-hour/week), individual history of the disease (including hypertension, hyperlipidemia, and diabetes [each yes or no]), energy intake (continuous; kcal/day), and SDS*.

e *Additionally adjusted for each other dietary pattern scores*.

## Discussion

This is the first study investigating how the dietary patterns derived by exploratory factor analysis are related to the CA in Chinese adults. These three dietary patterns were similar to those from other TCLSIH studies (28, 29). The results suggested that greater adherence to the sweets dietary pattern was positively related to CA in women aged 50 or older, whereas no relationships were observed between dietary patterns and CA in men.

From previous studies, we recognized that socio-demographic factors, lifestyle factors, nutritional status, and chronic diseases are related to CA (30, 31). Thus, we first adjusted for age and BMI. Second, we additionally adjusted for smoking status, alcohol consumption status, PA, total energy intake, education levels, employment status, household income, depressive symptoms, and personal and family history of diseases in model 3. After adjustments for these factors, the results indicated that the highest quartile of sweets dietary pattern scores was positively related to a higher prevalence of CA than those in the lower quartile in females aged 50 or older. Finally, we further adjusted for the other two dietary pattern scores in model 4. However, the results remained unchanged, suggesting that the sweets dietary pattern was related to CA independent of the other two dietary patterns.

Several studies were conducted on Western populations to investigate the relationship between empirically derived dietary patterns and CA. For example, in prospective investigations, the Mediterranean diet pattern, characterized by high intake of vegetables, legumes, fruits nuts, cereals, and olive oil, moderate intake of dairy products, red wine, fish, and low intake of saturated lipids (32), was associated with a lower risk of cardiovascular diseases (33, 34). However, to date, there was no consistent evidence indicating the relationship between the Mediterranean diet and CIMT progression (35, 36). The Western dietary pattern, characterized by high intake of saturated fat (processed red meat, French fries, margarine, and dairy products), trans fat (French fries and margarine), cholesterol (processed red meat), added sugar and low intake of fiber and micronutrients, similar to our Traditional Tianjin dietary pattern, has been shown to be positively related to subclinical arteriosclerosis in two prospective studies (15, 37). In contrast, another prospective cohort study conducted in 1,026 middle-aged French women did not observe the association between any dietary pattern and IMT or plaques (38). Differences in the regions, the definition of dietary patterns, variables adjustment, and participant characteristics may account for inconsistent results in literature.

In this study, we observed that high consumption of glycemic load foods (including fruits, pastry cake, sweets, snacks, etc) was positively related to CA in females age 50 years or older. This may be explained due to high intake of fruits or sweet foods with a high glycemic index has been shown to cause high glycemic load and insulin resistance, thereby leading to dyslipidemia, visceral fat accumulation, hypertension, endothelial cell activation, prethrombotic changes, and upregulation of inflammation levels, which have been considered potential causes of CA (39, 40).

Interestingly, such a positive relationship was not found in men. Our data showed that the scores of sweets dietary pattern in female participants (−0.74 to 1.12) are all higher than in male participants (−1.13 to 0.98) across increasing quartiles. This lower consumption of sweety food in men may contribute to the absence of an association between the sweets dietary pattern and CA in our study. In addition, male participants might have had a higher risk profile characterized by chronic inflammation and oxidative stress (41). The overall plaque burden and markers of inflammation appear to be greater in men than women (42), which may partially conceal the association between dietary patterns and CIMT in men. We also can not rule out the possibility of a statistical chance bias for the absence of an association in men.

According to our results, the traditional Tianjin dietary pattern defined in the current study, characterized by a high intake of animal liver, animal blood, seafood, freshwater fish, sea fish, and processed meat, was not related to CA. Vitamin B group contained in animal foods and long-chain n-3 polyunsaturated fatty acids contained in sea fish have been proven to improve CVD *via* homocysteine metabolism and reduction in serum triglycerides, which may offset the CVD risk caused by saturated fatty acids, trans-fatty acids, and dietary cholesterol intake in these animal foods (43, 44). The significant relationship between the health dietary pattern and CA disappeared after controlling for age and BMI, indicating that the relationship between dietary pattern and CA may be mediated in part through age and BMI. Our healthy pattern was characterized by high loading of vegetables. Usually, a vegetable diet is recognized as a healthy diet, which could be effective in cardiovascular prevention due to a higher intake of fiber, polyunsaturated fat, soy protein, plant sterols, antioxidants, and a lower intake of saturated fat (45, 46). However, gourd and root vegetables in this pattern (including pumpkin and potato) were highly related to carbohydrate intake, which is related to a greater progression of CA (47). All of these may offset its beneficial effect on CA (12, 48, 49). The exact biological mechanisms, underlying the putative effects of dietary patterns on CA risk, deserve more elucidation. Further studies based on objective biomarkers should be conducted to explore the gender difference and complex relationship between dietary patterns and CA.

The strengths of our study include a large sample size and the adjustments of various potential confounding factors, such as socio-demographic, clinically relevant variables, and lifestyle. However, several limitations must be listed. First, we could not infer causality between dietary patterns and CA due to the crosssectional design. Second, for FFQ, there was inevitably existing recall bias in self-reported questionnaires and seasonal changes in diet. In addition, some infrequently consumed foods might have been overlooked due to the limited food items on the FFQ. Although these may influence the accuracy of food intake, it indicated that dietary data were relatively stable over time according to our published studies (22, 50). Third, there may be some residual confounding factors we could not fully capture; variables that are intrinsically related to the dietary patterns may influence the relationship between dietary patterns and CA. More prospective cohort studies are warranted to test the relationship. Fourth, total variance explained by three dietary patterns was relatively low (17.3%), and other potential dietary patterns related to CA might not have been identified. Usually, the factors derived by factor analysis have a low to moderate proportion of intake explained, when a larger number of input variables are included in the procedure (51, 52). Aggregating similar food items into one food group is a way to improve variation of dietary patterns, which would lose detailed intake information. We used all 81 food items to conduct factor analyses to provide more detailed information about dietary patterns and generate the most distinctive dietary patterns of the study population, even though it resulted in a lesser total variance of dietary patterns. Finally, our study was based on Chinese adults aged 50 years or older, and the FFQ was specific and culturally appropriate for the local population. Thus, the application of results to other populations with different characteristics is limited.

## Conclusions

This is the first work to investigate the relationships between dietary patterns and CA based on Chinese. We found that greater adherence to sweets dietary patterns were positively related to CA in women aged 50 years or older. In addition, no relationship was found between any dietary pattern and CA in men. Further prospective studies are warranted to test this cross-sectional finding.

## Data Availability Statement

The raw data supporting the conclusions of this article will be made available by the authors, without undue reservation.

## Ethics Statement

The studies involving human participants were reviewed and approved by Medical Ethics Committee of the Tianjin Medical University with the reference number of TMUhMEC 201430. The patients/participants provided their written informed consent to participate in this study.

## Author Contributions

YL analyzed data and wrote the paper. YL, XuW, QZ, GM, LL, HW, YG, SZ, YW, TZ, MG, SS, XiW, MZ, QJ, and KS conducted research. LT and KN designed the research and had primary responsibility for the final content. All authors have read and approved the final manuscript.

## Funding

This study was supported by grants from the National Key Research and Development Project-Study on Diet and Nutrition Assessment and Intervention Technology (No. 2020YFC2006305), National Health Commission of China (No. SPSYYC 2020015), National Natural Science Foundation of China (Nos. 81941024, 91746205, 81872611, and 81673166), and 2014 and 2016 Chinese Nutrition Society (CNS) Nutrition Research Foundation–DSM Research Fund (Nos. 2016-046, 2014-071 and 2016-023).

## Conflict of Interest

The authors declare that the research was conducted in the absence of any commercial or financial relationships that could be construed as a potential conflict of interest.

## Publisher's Note

All claims expressed in this article are solely those of the authors and do not necessarily represent those of their affiliated organizations, or those of the publisher, the editors and the reviewers. Any product that may be evaluated in this article, or claim that may be made by its manufacturer, is not guaranteed or endorsed by the publisher.

## Abbreviations

BMI: body mass index
BP: blood pressure
CA: carotid atherosclerosis
CCA: common carotid artery
CI: confidence interval
CIMT: carotid intima-media thickness
CVD: cardiovascular disease
FBG: fasting blood glucose
FFQ: food frequency questionnaire
HDL-C: high-density lipoprotein cholesterol
LDL-C: low-density lipoprotein cholesterol
METs: metabolic equivalents
OR: odds ratio
PA: physical activity
SDS: Self-Rating Depression Scale
TC: total cholesterol
TCLSIH: Tianjin Chronic Low-grade Systemic Inflammation and Health
TG: triglycerides
WC: waist circumference
WDRs: weighed dietary record.
